# Supplementary material for: Novel Carboxylated Chitosan-Based Triptolide Conjugate for the Treatment of Rheumatoid Arthritis
Source: Pharmaceutics. 2020 Feb 26;12(3):202. doi: 10.3390/pharmaceutics12030202 (PMC7150988; doi:10.3390/pharmaceutics12030202)
Supplement: Supplementary file 1 [file pharmaceutics-12-00202-s001.pdf]

pharmaceutics

MDPI

# Supplementary Information: Novel Carboxylated Chitosan-Based Triptolide Conjugate for the Treatment of Rheumatoid Arthritis

Lan Zhang, Min Yan, Kun Chen, Qikang Tian, Junying Song, Zijuan Zhang, Zhishen Xie, Yong Yuan, Yaquan Jia, Xin Zhu, Zhenqiang Zhang, Xiangxiang Wu and Huahui Zeng

**$^{1}$H- and $^{13}$C-NMR spectra of all compounds:**

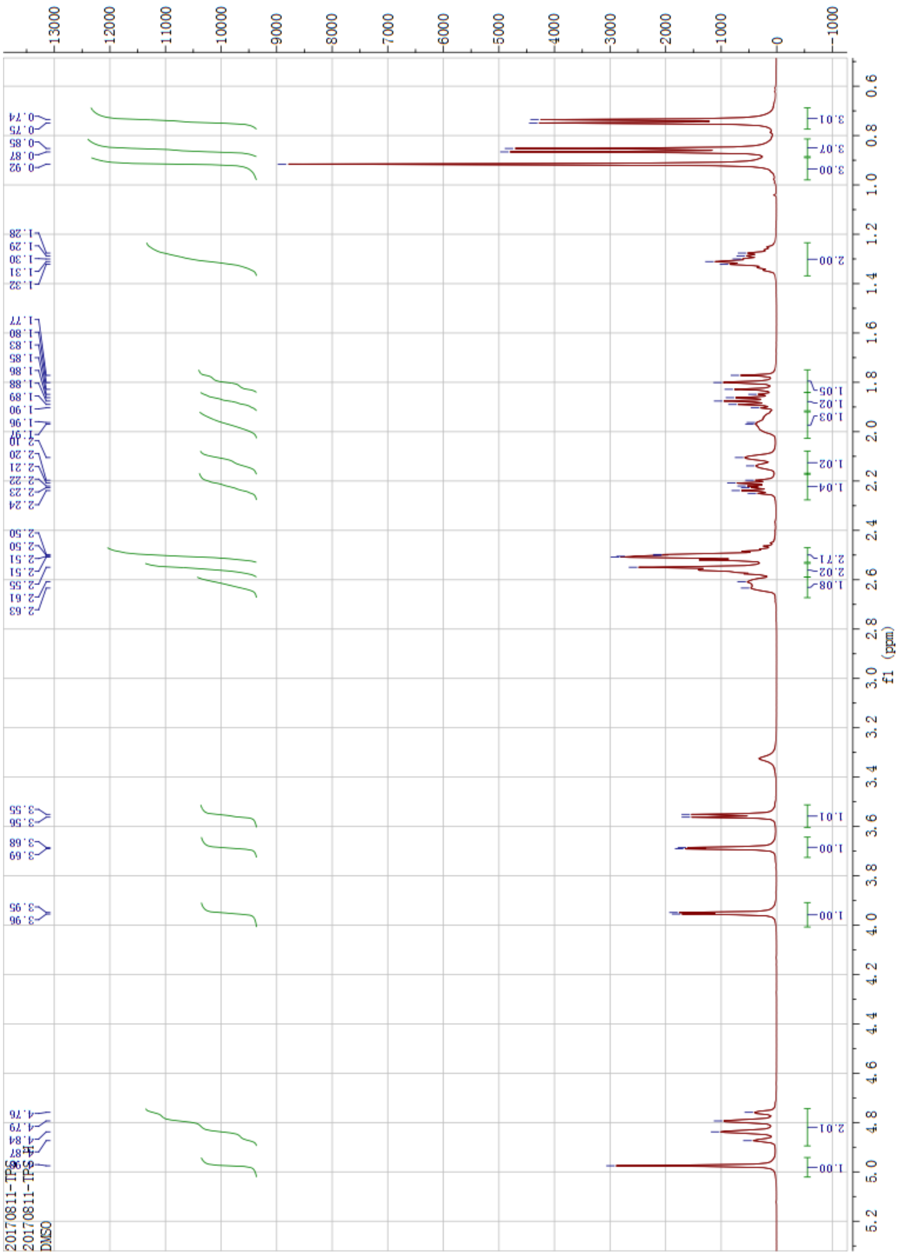

**Figure S1.** $^1$H spectra of **TPS**.

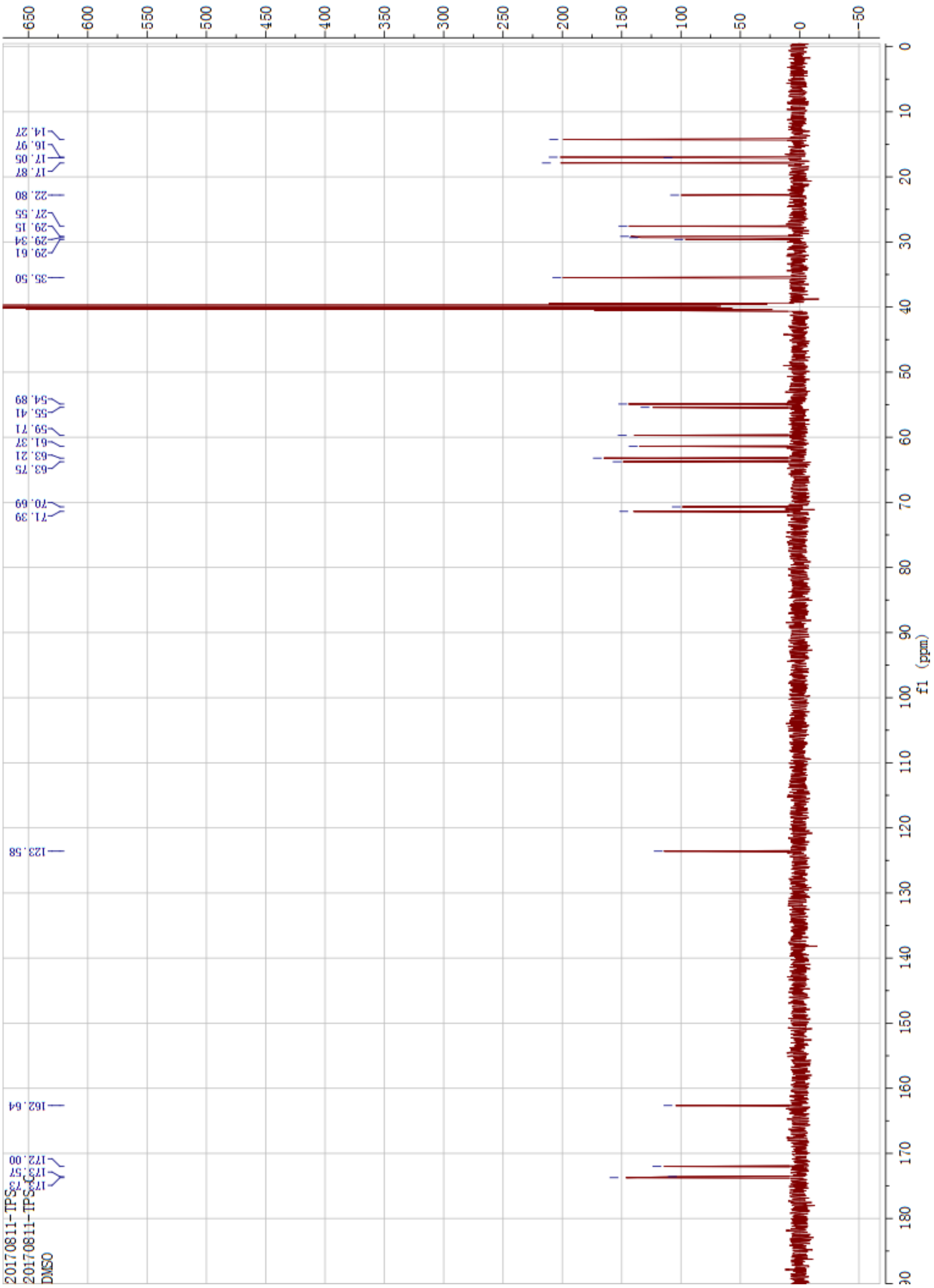


**Figure S2.** $^{13}C$ spectra of **TPS**.

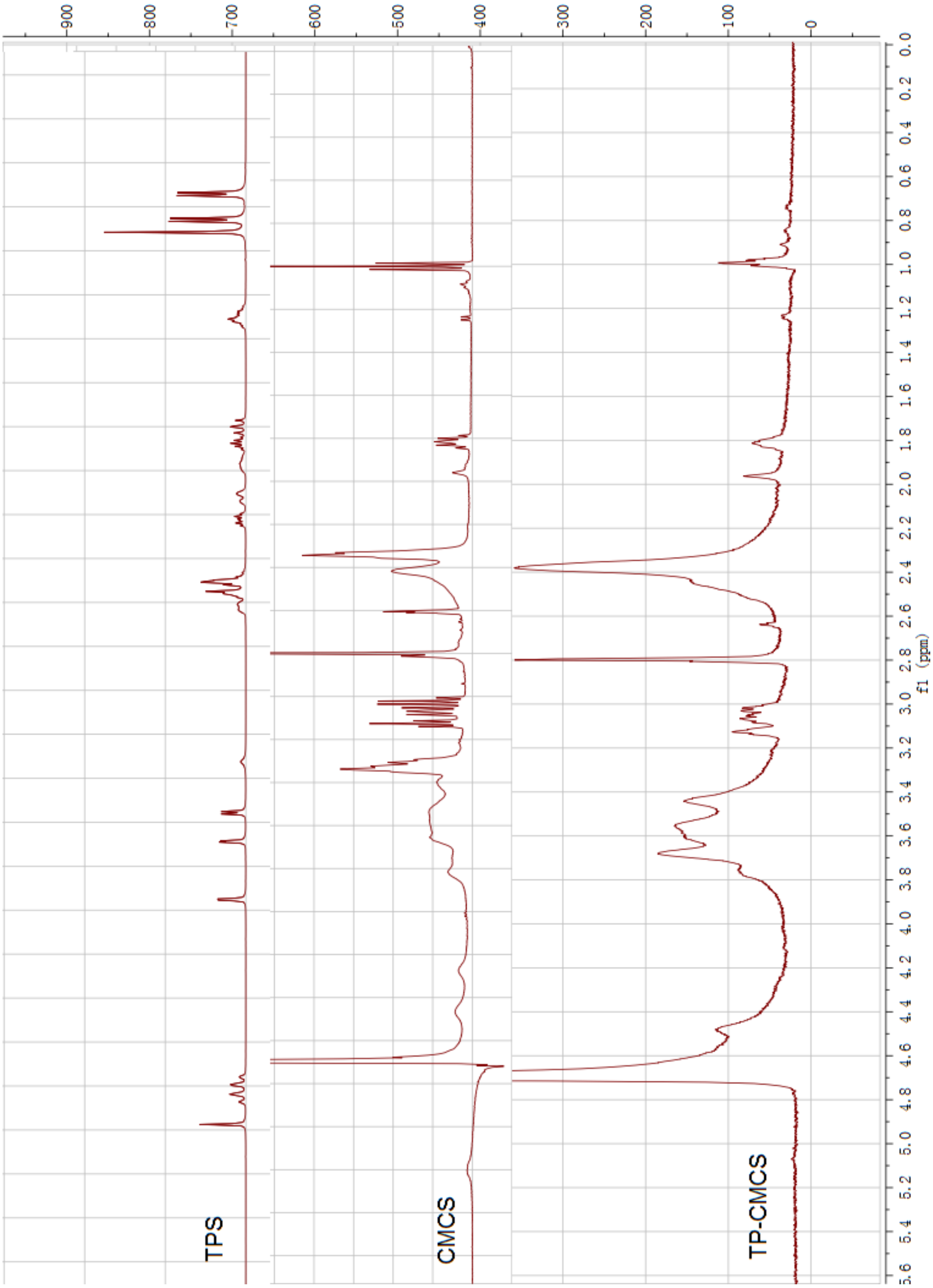


**Figure S3.** $^{1}$H spectra of **TP-CMCS**, **CMCS** and **TP**.

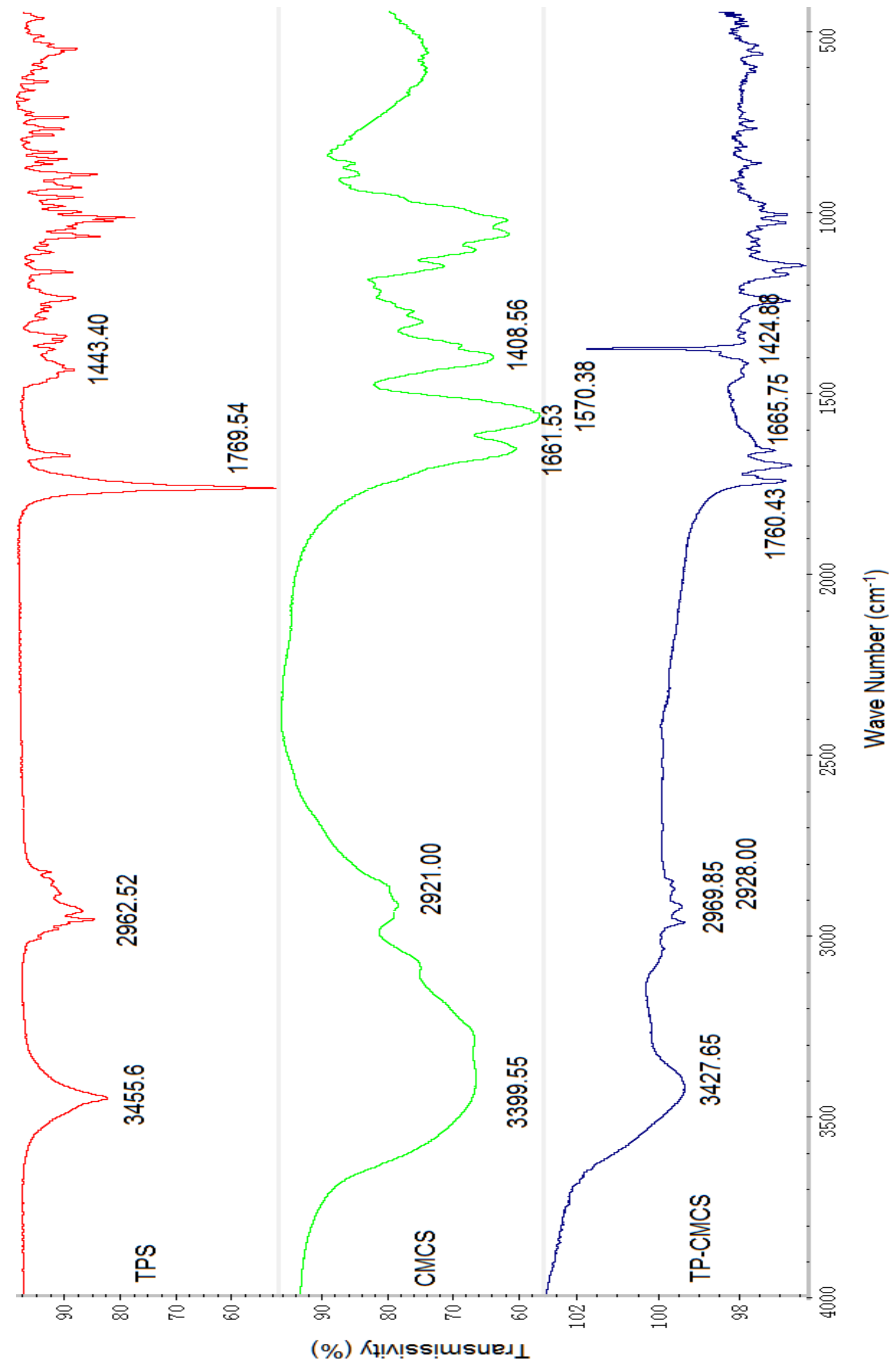


**Figure S4.** IR spectra of **TP-CMCS**, **CMCS** and **TP**.
